# Supplementary material for: Resolving the full spectrum of human genome variation using Linked-Reads
Source: Genome Res. 2019 Apr;29(4):635–45. doi: 10.1101/gr.234443.118 (PMC6442396; doi:10.1101/gr.234443.118)
Supplement: Supplemental Material [file supp_gr.234443.118_Supplemental_longranger-2.2.zip › longranger-2.2.tar.bz2!/lr2.2-public/lib/stan/cmdstan/stan/doc/api/html/classstan_1_1common_1_1recorder_1_1csv.html]

Stan: stan::common::recorder::csv Class Reference


|  |  |
| --- | --- |
|  | Stan  2.5.0  probability, sampling & optimization |


- Main Page
- Related Pages
- Namespaces
- Classes
- Files

- Class List
- Class Index
- Class Hierarchy
- Class Members

All Classes Namespaces Files Functions Variables Typedefs Enumerations Enumerator Friends Macros Pages

- stan
- common
- recorder
- csv

Public Member Functions |
List of all members

stan::common::recorder::csv Class Reference

Writes out a vector as string.
More...

`#include <csv.hpp>`

|  |  |
| --- | --- |
| Public Member Functions | |
|  | csv (std::ostream \*o, std::string prefix) |
|  | Construct an object. More... |
|  | |
| template<class T > | |
| void | operator() (const std::vector< T > &x) |
|  | Print vector as csv. More... |
|  | |
| void | operator() (const std::string x) |
|  | Print single string with a prefix. More... |
|  | |
| void | operator() () |
|  | Prints a blank line. More... |
|  | |
| bool | is\_recording () const |
|  | Indicator function for whether the instance is recording. More... |
|  | |

## Detailed Description

Writes out a vector as string.

Definition at line 19 of file csv.hpp.

## Constructor & Destructor Documentation

|  |  |  |  |
| --- | --- | --- | --- |
| stan::common::recorder::csv::csv | ( | std::ostream \* | *o*, |
|  |  | std::string | *prefix* |
|  | ) |  |  |

inline

Construct an object.

Parameters
:   |  |  |
    | --- | --- |
    | o | pointer to stream. Will accept 0. |

Definition at line 31 of file csv.hpp.

## Member Function Documentation

|  |  |  |  |  |
| --- | --- | --- | --- | --- |
| bool stan::common::recorder::csv::is\_recording | ( |  | ) | const |

inline

Indicator function for whether the instance is recording.

For this class, returns true if it has a stream.

Definition at line 89 of file csv.hpp.

template<class T >

|  |  |  |  |  |  |
| --- | --- | --- | --- | --- | --- |
| void stan::common::recorder::csv::operator() | ( | const std::vector< T > & | *x* | ) |  |

inline

Print vector as csv.

Uses the insertion operator to write out the elements as comma separated values, flushing the buffer after the line is complete

Template Parameters
:   |  |  |
    | --- | --- |
    | T | type of element |

Parameters
:   |  |  |
    | --- | --- |
    | x | vector of type T |

Definition at line 45 of file csv.hpp.

|  |  |  |  |  |  |
| --- | --- | --- | --- | --- | --- |
| void stan::common::recorder::csv::operator() | ( | const std::string | *x* | ) |  |

inline

Print single string with a prefix.

Uses the insertion operator to write out a string as comma separated values, flushing the buffer after the line is complete

Parameters
:   |  |  |
    | --- | --- |
    | x | string to print with prefix in front |

Definition at line 68 of file csv.hpp.

|  |  |  |  |  |
| --- | --- | --- | --- | --- |
| void stan::common::recorder::csv::operator() | ( |  | ) |  |

inline

Prints a blank line.

No prefix, no nothing.

Definition at line 78 of file csv.hpp.

---

The documentation for this class was generated from the following file:

- src/stan/common/recorder/csv.hpp


---

[ Stan Home Page ]

*© 2011–2014,
Stan Development Team.*
